# QSAR and Molecular Docking Studies of Oxadiazole-Ligated Pyrrole Derivatives as Enoyl-ACP (CoA) Reductase Inhibitors

**DOI:** 10.3797/scipharm.1310-05

**Published:** 2013-11-24

**Authors:** Kalyani D. Asgaonkar, Ganesh D. Mote, Trupti S. Chitre

**Affiliations:** AISSMS College of Pharmacy, Near R.T.O, Kennedy Road, Pune-411001, India.

**Keywords:** Antitubercular, Oxadiazole ligated pyrrole, *Mycobacterium tuberculosis*, Docking, Enoyl ACP co Reductase, 2D quantitative structure-activity relationship, 3D quantitative structure-activity relationship

## Abstract

A quantitative structure-activity relationship model was developed on a series of compounds containing oxadiazole-ligated pyrrole pharmacophore to identify key structural fragments required for anti-tubercular activity. Two-dimensional (2D) and three-dimensional (3D) QSAR studies were performed using multiple linear regression (MLR) analysis and k-nearest neighbour molecular field analysis (kNN-MFA), respectively. The developed QSAR models were found to be statistically significant with respect to training, cross-validation, and external validation. New chemical entities (NCEs) were designed based on the results of the 2D- and 3D-QSAR. NCEs were subjected to Lipinski’s screen to ensure the drug-like pharmacokinetic profile of the designed compounds in order to improve their bioavailability. Also, the binding ability of the NCEs with enoyl-ACP (CoA) reductase was assessed by docking.

## Introduction

Tuberculosis caused by *Mycobacterium tuberculosis* has become a global threat due to the emergence of resistant mycobacterium strains resulting in multiple drug-resistant tuberculosis (MDR-TB), extensive drug-resistant tuberculosis (XDR-TB), as well as total drug-resistant tuberculosis (TDR-TB) [1–3].

Of the different targets being explored in *Mycobacteria* for antitubercular activity, fatty acid synthesis inhibition is an attractive target for the rational design of new antitubercular agents. Mycolic acid is the major component of the *M. tuberculosis* cell wall. Enzymes that are responsible for fatty acid biosynthesis are considered as ideal targets for designing the new antimycobacterial agents. Fatty acid synthesis is catalyzed by fatty acid synthase enzymes-FAS-I and FAS-II. In mammals, the synthesis is catalyzed by FAS-I, whereas in *Mycobacterium* it is catalyzed by FAS-I and FAS-II. This difference renders FAS-II an attractive target for antitubercular discovery. The enoyl-ACP (CoA) reductase (FabI/ENR/InhA.) is an important enzyme in the FAS-II system [4]. In the *M. tuberculosis* inhA structural gene, (InhA) is the primary target of isoniazid, the most preferred anti-tubercular agent. InhA was identified as an NADH-dependent enoyl-ACP (CoA) reductase specific for chain elongation in precursors of mycolic acids [5].

Heterocycles possessing pyrrole are known to possess different biological activities like antibacterial, antitumor, analgesic, and anti-inflammatory along with antitubercular activity [6–11].

Some of the pyrrole derivatives are known to act as antitubercular by inhibiting the enzyme, FabI involved in fatty acid synthesis in *Mycobacterium* [4, 12–13].

Computational methods are an important tool in designing newer potent molecules [14]. These techniques have also been used to study pyrrole derivatives [15, 16].

As a continuation of our ongoing work on drug design and antimycobacterial studies [17], and to further explore the structural requirement for competitive inhibitors of enoyl-ACP (CoA) reductase, we herein report the molecular modeling studies on a series of pyrrole-ligated oxadiazole compounds synthesized by Rane et al [13].

Two-dimensional (2D) and three-dimensional (3D) quantitative structure-activity relation-ship (QSAR) studies were carried out. New chemical entities (NCEs) were then designed based upon the results of the 2D- QSAR and 3D-QSAR studies. Also, docking studies provided insight of the interaction of the compounds with the enzyme.

## Results and Discussion

Uni-Column Statistics revealed the observations (Tab. 1.):

1. The mean in the test set was found to be higher than the mean in the training set, indicating the presence of relatively more active molecules as compared to inactive ones.
2. A higher standard deviation in the training set indicates a wide distribution of activity of the molecules as compared to the test set molecules.

Descriptors that have shown either direct or indirect correlation with activity by more than 0.30 and intercorrelation less than 0.8 generated for the selected series of compounds have been considered (Tab. 2.).

### Interpretation of 2D-QSAR

Of the different methods carried out for 2D- QSAR, one of the best models was with 2D multiple linear regression (MLR) QSAR models and it showed the following statistical parameters: r^2^= 0.9827, cross-validated r^2^ i.e. q^2^= 0.5754 and parameter to assess external validation i.e. pred_r^2^= 0.8392 (Tab. 3). Descriptors such as chiV3Cluster, XKAverage, T_O_O_5, Rotatable Bond Count, SdsCHE-index were generated using the MLR method.

pMIC = + 6.6224 (chiV3Cluster) − 3.1570 (XKAverage) + 1.6748 (T_O_O_5) − 0.2851 (RotatableBondCount) + 0.0873 (SdsCHE-index)

**Fig. 1:**
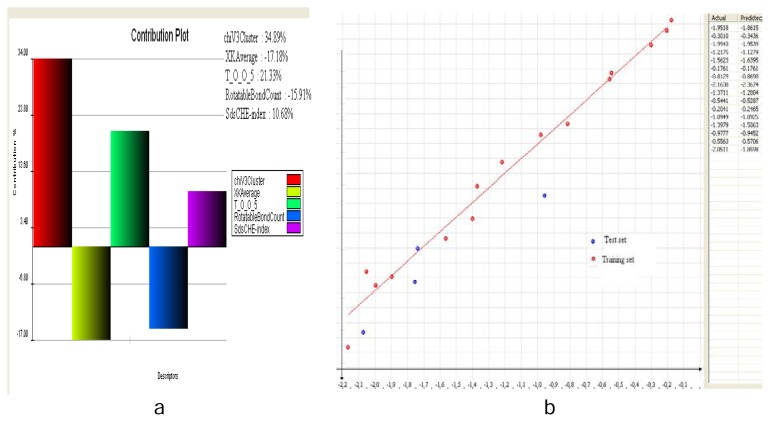
a: Contribution plot of selected descriptors. b: Plot of Actual versus predicted Activity

The above-mentioned descriptors showed the highest correlation with activity and also showed a proper distribution of data points (Fig. 1b). To increase the predictive power, different combinations of selected descriptors were tested by keeping T_O_O_5 as a constant descriptor. A careful observation of descriptors in the model (Fig. 1a) suggested that:

T_O_O_5 is an indicator variable which positively contributes to the QSAR equation up to 30% and signifies that the presence of an oxygen group at the R_1_ position of the ring is the most influential for ENR inhibitory activity. The descriptors like the sds CHE index indicates the number of −CH groups connected with one double bond and one single bond. Also, the chiV3 cluster signifies the valence molecular connectivity index of a third-order cluster. The other descriptors, XKAverage, Rotatable Bond Count, which are inversely proportional to activity, show that the average hydrophobicity value and rotatable bond count may be detrimental to biological activity.

### Interpretation of 3D- QSAR Model

**Fig. 2:**
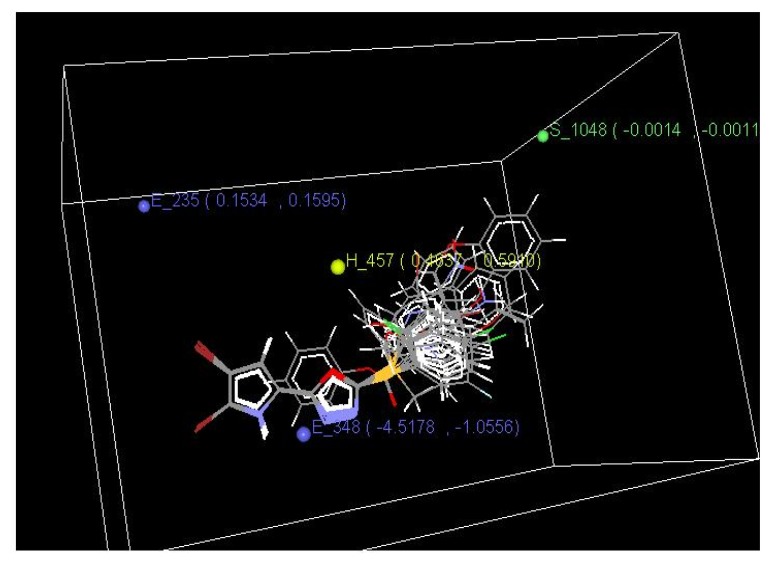
Common template data points generated using the kNN–MFA method (3D-QSAR) in a 3D rectangular grid showing contributions of electrostatic, hydrophobic, and steric functional groups for significant antitubercular activity.

3D-QSAR was used to optimize the electrostatic, steric, and hydrophobic requirements around the oxadiazole-ligated pyrrole pharmacophore. 3D data points were generated that contributed to the simulated annealing k-nearest neighbor molecular field analysis (SA kNN–MFA) 3D-QSAR model. The data points generated by 3D-QSAR are shown in Fig. 2.

The best model generated by the SA kNN-MFA method showed a q^2^, pred_r,^2^ and k-nearest neighbor as 0.5124, 0.7166, and 2, respectively (Tab. 4).

The ranges of data point values were based on the variation of the field values at the chosen points using the most active molecule and its nearest neighbor set. The points generated in the SA kNN–MFA 3D-QSAR model were S_1048, H_457, E_348, E_235 i.e. steric, hydrophobic, and electronic data points at lattice points 1048, 457, 348, and 235, respectively. Negative steric values indicated that the less steric groups were required to increase activity. Similarly positive and negative values in the electrostatic field descriptors indicated the requirement of electropositive and electronegative electrostatic potential, respectively, for enhancing the biological activity of oxadiazole-ligated pyrrole pharmaco-phore derivatives.

**Fig. 3:**
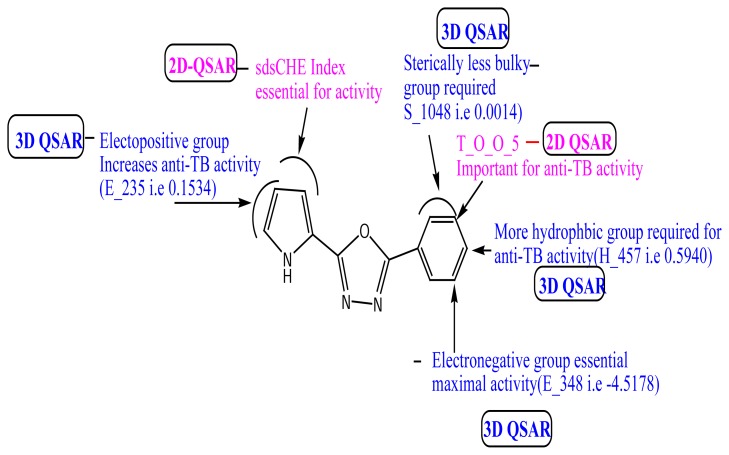
Pharmacophoric requirements around pyrrole-ligated oxadiazole derivatives

Based on the results, the 2D and 3D QSAR pharmacophoric requirements for oxadiazole-ligated pyrrole pharmacophore are compiled in Fig. 3.

### Design of New Chemical Entities (NCEs) Containing Pyrrole-Ligated Oxadiazole Pharmacophore

The pharmacophore optimizing of pyrrole-ligated oxadiazole and designing NCEs to have potent antitubercular activity was done based on the results of 2D- and 3D-QSAR studies. All of the designed NCEs (Tab. 5) showed a Lipinski score of 6 and the predicted activity was between the most potent and least potent compound of the reported series.

### Docking Studies

Docking studies helped to sort out the designed compounds with good binding affinity against the enoyl-ACP (CoA) reductase enzyme (ENR). The docking score in terms of the GLIDE score (G-score), the results of the docking studies of the designed compounds of oxadiazole-ligated pyrrole series, are presented in Tab. 6.

### G-score

The scoring function of the GLIDE docking program is presented in the G-score form. A G-score indicates the binding affinity of the designed compound to the receptor/enzyme. The G-scores of the designed NCEs 1 and 11 were found to be −7.099278 and −7.09647, respectively, and were comparable with the G-score of the standard drug i.e isoniazid (−7.500947).

### H-Bond Interactions

The H-bond is one of the most widely used parameters for the evaluation of the docking results, as it is an influential parameter in the activity of the drug compound. The number of H-bond interactions in the standard compounds was compared with that of the designed NCEs. The number of H-bond contacts for the designed compounds 1–6, 8–10, and 12 was found to be one, and compound 11 showed two hydrogen bonds as compared to the standard (isoniazid) which showed three hydrogen bonds.

### Contacts

The contacts are represented in the form of van der Waals (vdw) interactions as good vdw interactions, bad vdw interactions, and ugly vdw interactions.

It was found that all of the designed compounds had a higher number of good vdw, bad vdw, and ugly vdw interactions when compared with the standard isoniazid. However, the G-scores for these molecules were lower. In conclusion, the G-score and H-bond interactions, and the number of good, bad, and ugly vdw contacts decided the possible binding affinity and in turn potency of the designed NCEs.

**Fig. 4:**
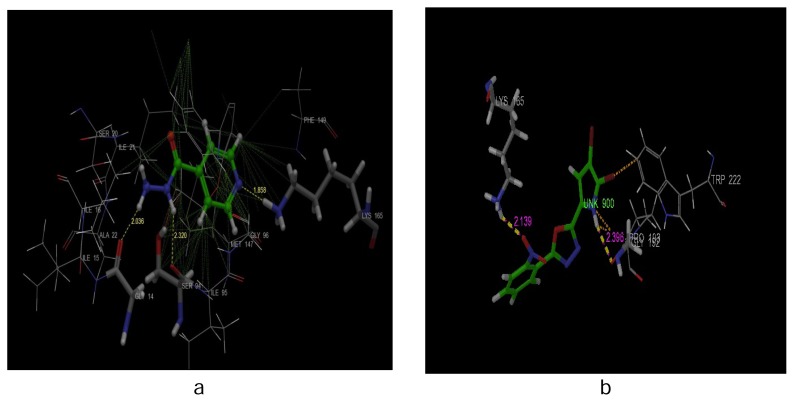
Docking interaction of (a) isoniazid with Enoyl-ACP (CoA) reductase and (b) Interaction of compound 11 with Enoyl-ACP (CoA) reductase

Isoniazid showed three hydrogen bonds viz, nitrogen of the pyridine ring with lysine (Lys165, 1.858 Å), tertiary N of the hydrazide moiety with serine (Ser94, 2.32 Å), and primary N of the hydrazide moiety with glycine (Gly14, 2.036 Å) (Fig. 4).

Docking studies showed that the designed compounds and standard bond in the same binding pocket contained the amino acids Lys165 and Gly14. The bromo substituent on the pyrrole nucleus formed hydrophobic bonds with isoleucine (Ile 21), serine (Ser20), and tryptophan (Trp 222). The nitrogen of pyrrole has shown the hydrogen bond with Gly14. Compound 11 showed the H-bond interaction with the Lys165 residue (Fig. 4). The NO atom on the benzene ring of the pyrrole-ligated oxadiazole nucleus showed the H-bond interaction with the NH group of Lys165 (2.139 Å).

## Materials and Methods

### Data Set

A data set (20 molecules) of oxadiazole-ligated pyrrole derivatives with varied chemical and biological activities, reported by Rajesh Rane et al. for antimycobacterial activity, was considered for the molecular modeling studies [13]. Biological activity expressed in minimum inhibitory activities (MIC) was converted into the corresponding pMIC (pMIC = −log(MIC) values. The structures and antimycobacterial activity of the molecules are given in Tab. 7.

### Computational Details

All the computational studies were carried out using the V-Life sciences, MOLECULAR DESIGN SUITE (MDS) version 3.5 [18].

All the computational molecules were drawn in Chem. Draw Ultra 8.0 and geometry optimization was done using the standard Merck molecular force field (MMFF) with distance-dependent dielectric function and energy gradient of 0.001 kcal/mol Å. The geometry of each molecule was further optimized with the MOPAC 6 package using the semi-empirical AM1 Hamiltonian. The initial conformations were selected and minimized using the Powell method until the root-mean-square deviation 0.001 kcal/mol Å was achieved [19, 20].

### Experimental Design

The dataset of 20 molecules was divided into the training and test sets using the random selection method. Random selection is a technique by which all compounds are divided into a training set and test set in a specific ratio or percentage. The training and test set should be representative of the entire data set, hence while dividing the test and training sets the majority of the molecules should be in the training set, so usually the preferred percentage of molecules in the training set is above 70%. Hence in the present study, 80% in the training set and 20% in the test set was adopted. By the random selection method, 20 molecules were divided into the training set (16 molecules) and test set (4 molecules). In an attempt to ensure the robustness of the model and increase the predictive ability of the QSAR model, they were subjected to a randomization test. It was ensured that representative points in the test set were close to those of the training set and vice versa and the training set showed chemical and biological diversity [18].

### Uni-Column Statistics

Uniform representation of the molecules in the training and test sets was confirmed through uni-column statistics. It was observed that the maximum value of the pMIC50 of the test set was less than or equal to the maximum value of the pMIC50 of the training set, and the minimum value of the pMIC50 of the test set was higher than or equal to the minimum value of the pMIC50 of the training set, indicating that the test set was interpolative and derived within the minimum-maximum range of the training set. Values for the mean and standard deviation pMIC50 of the training and test sets indicate a relative difference of the mean and point density distribution of the two sets [17, 21].

### 2D-QSAR

Different models were generated for the 2D-QSAR study using MLR with simulated annealing as the variable selection method [21–23].

Various 2D descriptors like topological, physicochemical, alignment-independent, and atom-type count descriptors were calculated after about 100 independent descriptors were processed by removing the invariable column. Further refinement in the selection of descriptors has been carried out using the correlation matrix, to obtain the most representative descriptors [24–26].

### 3D-QSAR MODEL

The selected series of compounds were aligned using the template-based alignment method (Fig. 2) and the resulting set of aligned molecules was then used to build the 3D- QSAR models. In the template-based alignment method, pharmacophore is first selected and its template is drawn. On the chosen template, all the molecules are then subjected to alignment. The molecules that are not aligned due to mismatch of the template or any other reason are not considered for alignment [18].

The training and test sets were selected by random selection in the range of 80%. Regression was done by the SA-kNN method implementing leave-one-out (LOO) cross-validation [21–23]. Leave-one-out (LOO) cross-validation is one of the simplest procedures for model validation. It consists of removing each sample once and a new model is created for the remaining samples. Thus if there are N number of samples, LOO is done N times, generating predicted values for each number of factors. The LOO approach changes the data structure by removing 1/Nth compound in each cross-validation turn leading to an increasingly smaller perturbation with increasing N. The differences between the experimental and estimated values from the model are used to calculate the root mean square error of the cross-validation (RMSECV) and the correlation coefficient of leave-one-out cross-validation for the training set.

The 3D descriptors were calculated as electrostatic, steric, and hydrophobic [23, 27].

Many models were generated, but the best model satisfied all of the following statistical parameters:

n, number of molecules (>20 molecules);
k, number of descriptors in a model (statistically n / 5 descriptors in a model);
df, degree of freedom (n – k −1) (higher is better);
r^2^, square of regression (>0.7);
q^2^, cross-validated r^2^(>0.5);
pred_r^2^ for external test set (>0.5);
SEE, standard error of estimate (smaller is better);
F-test, F-test for statistical significance of the model (higher is better, for the same set of descriptors and compounds);
F_prob. Alpha – error probability (smaller is better);
Z score, calculated by the randomization test (higher is better);
best_ran_q^2^, highest q^2^ value in the randomization test (as low as compared to q^2^);
best_ran_r^2^, highest r^2^ value in the randomization test (as low as compared to r^2^);
a- statistical significance parameter by randomization test (<0.01)

In the kNN method, an unknown member is classified according to the majority of its k-nearest neighbors in the training set. In this method, the activity of each compound is predicted as an average activity of k most chemically similar compounds from that data set. If the residual values obtained by the subtraction of the predicted activities from the biological activities are toward zero, the model is said to have a good predictive ability. The plots of observed versus predicted activities of both training and test set molecules helped in the cross-validation of the kNN-QSAR model [14, 17].

### Model Validation

To test the stability and predictive ability of the developed QSAR models, the models were validated using internal validation, external validation, and randomization test. The LOO method was used to validate all of the models generated by 2D and 3D-QSAR.

### Internal Validation

This was carried out to check whether the training and test set molecules were properly distributed. All cross-validation studies were performed by considering the fact that a value of q^2^ is > 0.5 and r^2^ > 0.7 (r^2^ is an indication of training set and q^2^ is an indication of cross-validated r^2^, i.e. test set molecules).

### External Validation

External validation of the generated models was carried out by predicting the activity of the test set of the compounds. This was done by considering the value of pred_r^2^, which should be above 0.5.

The generated models were found to have values in the required range.

### Randomization Test

This is the most popular tool used by researchers to prevent chance correlation. The models generated were compared with a random data set obtained by rearranging the activities in the training set. After each permutation, r^2^ and q^2^ were recorded. If in each case the r^2^ and q^2^ gave very low values compared to the original data, then we can say with some confidence that the original QSAR model was not generated by chance. The best_ran_q^2^, highest q^2^ value in the randomization test, was low compared to q^2^; best_ran_r^2^, highest r^2^ value in the randomization test, was low compared to r^2^; the Z-score gave the statistical significance of the model (<0.01).

The standard error of estimate (SEE) was also considered before selecting a particular model [14, 17].

### Designing of New Chemical Entities (NCEs)

Based on 2D-QSAR and 3D-QSAR, the NCEs that would follow Lipinski’s rule were designed using the LEADGROW tool. [21, 28]. These designed NCEs were then subjected to docking studies.

### Docking Studies

The molecular docking tool, Glide (Schrödinger, LLC, New York) software was used for studying the binding modes of the designed compounds into the binding pocket of enoyl-ACP (CoA) reductase enzyme (ENR).

The crystal structures of ENR were obtained from a protein databank (PDB Code2IDZ). All structures were prepared for docking using ‘protein preparation wizard’ in Maestro Wizard 8.5.

The final evaluation was done with glide score (docking score) and the single best pose was generated as the output for the particular ligand.

$$\text{Gscore}=\text{a }\times \;\text{vdw}+{\text{b}}^{*}\;\text{cow}\;ϸ\;\text{Lipo}+\text{H bond}+\text{Metal}+\text{BuryP}+\text{Rot B}+\text{Site}$$

where, vdW, van der Waal energy; Coul, Coulomb energy; Lipo, lipophilic contact term; H Bond, hydrogen-bonding term; Metal, metal-binding term; Bury P, penalty for buried polar groups; RotB, penalty for freezing rotatable bonds; Site, polar interactions at the active site; and the coefficients of vdW and Coul are: a = 0.065, b = 0.130.

## Conclusion

The present study was focused on the development of the potential compound containing the pyrrole-ligated oxadiazole analogue with anti-TB activity using QSAR studies. 2D- and 3D-QSAR results shed light on the electronic, steric, hydrophobic, and topological nature of the substitution pattern around the selected pyrrole-ligated oxadiazole pharmacophore. The 2D-QSAR study indicated the requirement of T_O_O_5 and the sds CHE index which positively contributed to the biological activity. 3D-QSAR gave information about the nature of the substituents like the electron-withdrawing group at the 4^th^ and 5^th^ position of pyrrole, the less steric group at meta, para, and ortho position on the benzene ring, the electron-withdrawing group at the 4^th^ position of benzene, and finally the more hydrophobic group at para position of the benzene ring is required for good antimycobacterial activity. The designed compounds were subjected to Lipinski’s filter, which gave information about the pharmacokinetic behavior. The designed compounds also showed a good binding interaction with the enoyl-ACP (CoA) reductase enzyme.

The correctness of the rationale behind these dry lab studies can be further validated by carrying out the synthesis and antitubercular activity of the designed NCEs.

## Figures and Tables

**Tab. 1:** Uni-Column statistics for the training set and test set.

Model-1	Column name	Average	Max	Min	Std.dev.	Sum
Training	pMIC	−1.1986	−0.1761	−2.1638	0.7035	−19.1770
Test	pMIC	−1.6286	−0.9542	−2.0719	0.4754	−6.5146

**Tab. 2:** Correlation matrix

Descriptor	chiV3Cluster	XKAverage	T_O_O_5	Rotatable Bond Count	SdsCHE-index
chiV3Cluster	1	−0.4728	−0.61661	−0.63246	0.3423
XKAverage	−0.4728	1	0.71216	0.726289	0.444362
T_O_O_5	−0.61661	0.71216	1	0.729458	0.553539
Rotatable Bond Count	−0.63246	0.726289	0.729458	1	0.5
SdsCHE-index	0.3423	0.444362	0.553539	0.5	1
pMIC	0.9387	0.6475	−0.1257	0.2749	0.4148

**Tab. 3:** Statistical results of 2D- QSAR generated by MLR

Statistical Parameter	2D- QSAR MLR analysis values	Contributing descriptors
n	16	chiV3Cluster
r^2^	0.9827	XKAverage
r^2^ se	0.1134	T_O_O_5
q^2^	0.5754	RotatableBondCount
q^2^ se	0.3615	SdsCHE-index
F test	113.3738	
pred_r^2^	0.8392	
pred_r^2^se	0.2757	

**Tab. 4:** Statistical results of 3D- QSAR generated by SA kNN-MFA

Statistical parameter	3D-QSAR SA-kNN-MFA
q^2^	0.5124
q^2^ se	0.3647
Pred_r^2^	0.7166
Pred_r^2^ se	0.4207
N	15
K nearest neighbor	2
Contributing descriptors	S_1048, H_457, E_348, E_235

**Tab. 5:** Structures of designed NCEs.

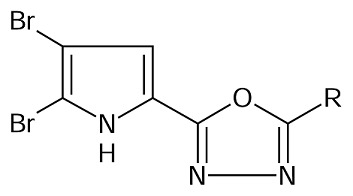			

Cpd. no.	R	Cpd. no.	R
1	3-chlorophenyl	7	3-ethylphenyl
2	3-bromophenyl	8	3-methoxyphenyl
3	3-iodophenyl	9	4-methoxyphenyl
4	4-methylphenyl	10	2-methoxyphenyl
5	4-ethylphenyl	11	2-nitrophenyl
6	3-methylphenyl	12	3-nitrophenyl

**Tab. 6:** Results of docking studies of designed compounds of oxadiazole-ligated pyrrole series

Cpd. No.	G-Score	Hydrogen bonds	Good vdw	Bad vdw	ugly
**1**	−7.099278	1	224	0	0
**2**	−6.833546	1	226	0	0
**3**	−6.538829	1	239	3	0
**4**	−6.440214	1	220	7	0
**5**	−6.382636	1	214	2	0
**6**	−6.293452	1	197	3	0
**7**	−6.146766	0	235	5	0
**8**	−6.86602	1	238	9	0
**9**	−6.05158	1	242	7	0
**10**	−6.96048	1	246	8	0
**11**	−7.09647	2	196	3	0
**12**	−6.05158	1	207	1	1
**Isoniazid**	−7.500947	3	155	0	0

**Tab. 7:** Selected series of oxadiazole-ligated pyrrole derivatives

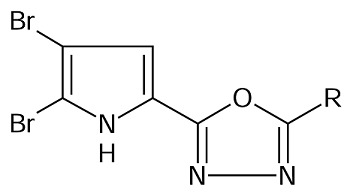			

Cpd. No.	R	MIC(μg/ml)	pMIC
5a	2-hydroxyphenyl-	36.50	−1.56229
5b	phenylacetic-	145.80	−2.16376
5c	4-aminophenyl-	16.50	−1.21748
5d	4-chlorophenyl-	9.50	−0.97772
5e*	4-methoxyphenyl-	56.50	−1.75205
5f	2,4-dichlorphenyl-	1.60	−0.20412
5g	2-phenylethenyl	78.50	−1.89487
5h	4-hydroxyphenyl-	25.00	−1.39794
5i	5-flouro-2-chlorphenyl-	6.50	−0.81291
5j*	4-nitrophenyl-	9.00	−0.95424
5k	4,5-dibromo-1H-pyrrol-2-yl-	3.50	−0.54407
5l	phenyl-	98.70	−1.99432
5m	2-methoxy-4-vinylphenoyl-	112.50	−2.05115
5n	Pyridine-4-yl	3.50	−0.5563
5o	4H-chromen-3-yl-vinyl-	2.00	−0.30103
6*	-SH	54.50	−1.7364
7a	methyl-S-	89.50	−1.95182
7b*	ethyl-S-	118.00	−2.07188
7c	phenyl-S-	23.50	−1.37107
7d	acetophenone-S-	1.50	−0.17609

* indicates test set; pMIC = −logMIC
